# Mesenchymal stem/stromal cell therapy in atopic dermatitis and chronic urticaria: immunological and clinical viewpoints

**DOI:** 10.1186/s13287-021-02583-4

**Published:** 2021-10-11

**Authors:** Eun-Young Kim, Hyuk Soon Kim, Ki-Sung Hong, Hyung-Min Chung, Se-Pill Park, Geunwoong Noh

**Affiliations:** 1 Miraecellbio Co., Ltd., Seoul, Korea; 2 Department of Biomedical Sciences, College of Natural Science, The Graduate School of Dong-A University, Busan, Korea; 3 Department of Health Sciences, The Graduate School of Dong-A University, Busan, Korea; 4 Department of Stem Cell Biology, School of Medicine, Konkuk University, Seoul, Korea; 5 Faculty of Biotechnology, College of Applied Life Sciences, Jeju National University, Jeju, 63243 Korea; 6 Department of Allergy, Allergy and Clinical Immunology Center, Cheju Halla General Hospital, Doreongno 65, Jeju-si, 63127 Jeju Special Self-Governing Province Korea

**Keywords:** Mesenchymal stem/stromal cell, Atopic dermatitis, Chronic urticaria, Clinical viewpoint, Allergy

## Abstract

Allergic diseases are immune-mediated diseases. Allergies share a common immunopathogenesis, with specific differences according to the specific disease. Mesenchymal stem/stromal cells (MSCs) have been applied to people suffering from allergic and many other diseases. In this review, the immunologic roles of MSCs are systemically reviewed according to disease immunopathogenesis from a clinical viewpoint. MSCs seem to be a promising therapeutic modality not only as symptomatic treatments but also as causative and even preventive treatments for allergic diseases, including atopic dermatitis and chronic urticaria.

## Introduction

Allergic diseases are immune-mediated diseases. Allergic asthma, allergic skin diseases, allergic rhinitis and allergic conjunctivitis are the most prevalent allergic diseases [1]. The general common immunopathogenesis is Th1/Th2 imbalance [2], but the specifics differ according to the allergic diseases.

The main treatments for allergic disease are symptomatic treatments including corticosteroids, antihistamines and antileukotrienes, which temporarily inhibit inflammatory mediators and immune cells [3]. Causative treatments such as desensitization and tolerance induction have also been applied [4]. However, patients suffer from symptoms and signs due to the repetitive recurrence of diseases and the continuous medication. Cyclosporin A and alkylating agents are used for refractory allergic diseases [5]. Some patients want to end their need for medication by achieving remission or cure through causative treatment. Sometimes patients meet conditions in which they cannot take medication, such as pregnancy, and various adverse effects can occur after long-term treatment. A new therapeutic modality may be needed for the effective treatment of allergic diseases.

Mesenchymal stem/stromal cells (MSCs) are the major stem cells in the field of cell therapy [1]. MSCs have been applied clinically for more than 10 years and have been proven to be safe and effective for autoimmune and inflammatory disorders. Recently, mesenchymal cell therapy (MSCT) has been tried to treat allergic diseases. Atopic dermatitis (AD) and chronic urticaria (CU) are representative systemic allergic skin diseases [6]. Although skin eruption and itching are representative symptoms and signs in AD and CU, the forms of skin eruption are completely different in these two diseases. Eczematous lesions are the typical form of skin lesions in AD, while wheals, hives and/or angioedema are the characteristic skin eruptions of CU, and these have different immunopathogeneses [7, 8].

This article reviews MSCT relating to the pathogenesis of AD and CU as a new therapeutic modality from the clinical viewpoint of the immunopathogenesis of allergic disease by reconstructing the immunogenesis according to clinical aspects and matching the relevant immunologic roles of MSCs.

## General immunopathogenesis of allergic diseases from a clinical viewpoint

Immune reactions are classified into four subtypes by the original Gell and Coombs classification categories: type 1, immediate or IgE mediated; type II, cytotoxic or IgG/IgM mediated; type III, IgG/IgM immune complex mediated; and type IV, delayed-type hypersensitivity or T-cell mediated [9]. Type II immune reactions are also subclassified into type IIa for the cytotoxic type and type IIb for the antibody-mediated cell-stimulating type. Type IV immune reaction is also subclassified into type IVa for CD4+ Th1 cell mediated with activation of macrophages, type IVb for CD4+ Th2 cell mediated with eosinophilic involvement, type IVc for cytotoxic CD8+ T cell with involvement of perforin-granzyme B in apoptosis and type IVd for T-cell-driven neutrophilic inflammation [10]. The immunopathogenesis of AD and CU is not simple. Immunoglobally, AD shows type I and type IV2 immune reactions, and CU consists of type 1, type IIb [11] and/or type III immune reactions (Table 1). In CU, the immune reactions by autoantibodies to IgE are possibly type I and somewhat similar to type III immune reactions, but they are stimulated as type 1 reactions rather than serum sickness or inflammation of binding cells by immune complexes.

**Table 1:** The types of immune reactions in the key immunopathogeneses of atopic dermatitis and chronic urticaria

Allergic disease	Key immunopathogenesis	Type of immune rections
Atopic dermatitis	Allergen-specific IgE	Type I
	Allergen-specific Th2 cell	Type Ivb
Chronic urticaria	Specific IgE for autoantigens	Type I
	Autoantibody for FcεR1	Type IIb
	Autoantibody for IgE	Type I/Type III style

**Fig. 1:**
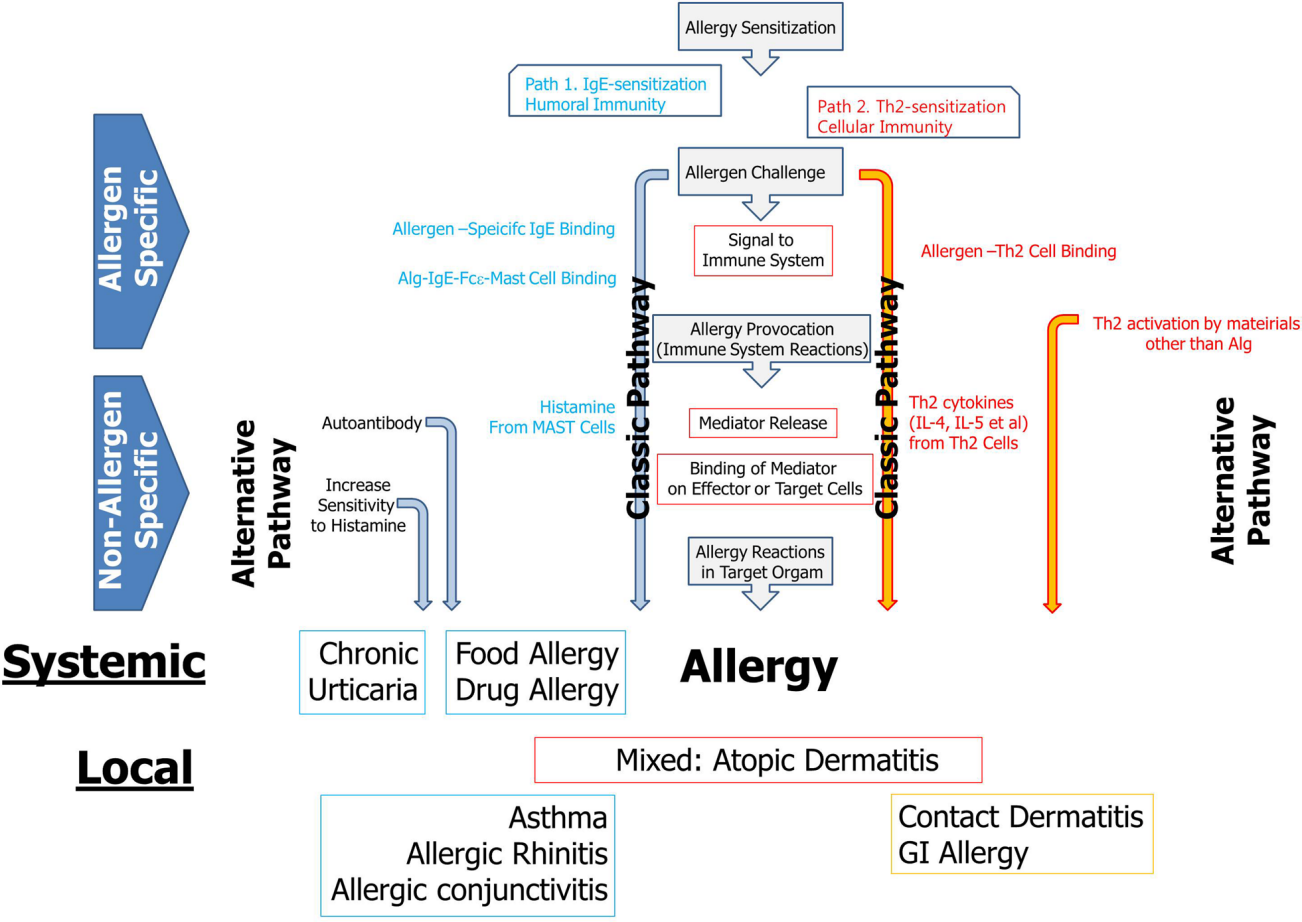
The structure of the immunopathogenesis of allergic diseases. First, allergen-specific IgE and/or allergen-specific Th2 cell responses manifest through the sensitization phase. Thereafter, the clinical manifestations develop by allergy provocation through rechallenge with sensitized allergens. The classical pathway of immunopathogenesis of IgE-mediated allergy consists of allergen-IgE-FcεR1 binding on mast cells, followed by histamine release. Finally, histamine provokes allergic symptoms and signs, including urticaria, dyspnoea and even anaphylaxis. Additionally, there is an allergen-specific Th2 cell-mediated pathway that is important in eosinophilic inflammation in atopic dermatitis. Classically, many allergic diseases are allergen-specific. In chronic urticaria, autoimmune mechanisms play a role as an alternative pathway of immunopathogenesis and are not allergen-specific. Anaphylaxis due to drug allergies is a systemic disease, whereas allergic rhinitis and allergic conjunctivitis are generally limited to the nose and eyes. Allergic diseases present as local or systemic diseases

In this review, the immunopathogenesis of allergic disease is approached according to the type of immune response and the effector mechanism from the clinical viewpoint. The general common immunopathogenesis is Th1/Th2 imbalance [2]. From this basic immunologic status, allergens stimulate the sensitization phase and effector processes [12]. Classically, the immunopathogenesis of allergic diseases from the sensitization phase and effector phase consists of two pathways (Fig. 1). One is IgE-mediated (humoral), and the other is Th2 cell-mediated (cell mediated) when the dominant status is Th2. In sensitization processes, allergens acquire allergen-specific IgE and/or allergen-specific Th2 cells. However, allergen-specific tolerance can also be achieved during the sensitization phase.

Once allergen-specific IgE and/or allergen-specific Th2 cells are acquired without the acquisition of allergen-specific tolerance in the sensitization phase, patients show clinical symptoms and signs of rechallenged allergens thereafter through the effector processes [12]. In the effector processes, two immunopathogenic pathways, through allergen-specific IgE and allergen-specific Th2 cells, lead to clinical symptoms and signs that result in the corresponding allergic disease.

IgE-mediated diseases follow the sequence of allergen binding to a specific IgE; the IgE binding to mast cells expressing the representative IgE receptor; granulation of mediators such as histamine, which is the representative mediator in the IgE-mediated allergic disease process in mast cells; and histamine binding to the cells expressing histamine receptor [12]. Finally, allergic symptoms and signs develop, such as urticaria and anaphylaxis, which are mediated through drug-specific IgE, and food allergen-specific IgE, rhinitis and asthma, which are mediated through allergens such as house dust mites, pollen, fungi and animal furs, as well as drugs and food allergens.

Th2-mediated disease begins with Th2 cytokine production, including IL-4 and IL-5, which mediate IgE production and eosinophilic inflammation [12]. Through this immunopathogenesis, eosinophilic inflammation in the affected organ occurs as AD. In the case of AD, the characteristic skin manifestations are eczematous lesions that differ completely from urticaria or angioedema in CU, as an IgE-mediated disease.

Many immunopathogeneses have been reported. Briefly, AD is a representative complicated allergic disease that is mediated through Th2 cells and the resultant allergic skin inflammation as a form of generalized or local eczema [7]. Generalized or local wheals and/or angioedema are the characteristic skin eruptions of CU, which are mediated by histamine that is produced through the IgE-FcRε-MAST cell granulation-histamine pathway [8].

Concerning the allergenic causes of these diseases, allergic diseases are generally allergen-specific [12]. Therefore, it is known that specific allergens provoke allergic reactions. However, no allergenic cause has been identified in some allergic diseases that are well known as allergen-specific. Moreover, the aetiology of CU is unknown, and even autoimmune mechanisms have been suggested [8]. Besnier’s prurigo is a kind of AD. Laboratory tests are basically normal in Besnier’s prurigo [13]. Some authors have described CU and Besnier’s prurigo as non-allergen-specific allergic diseases.

The basic concept of the immunopathogenesis of allergic disease revolves around histamine, which is released from mast cells through allergen-specific IgE binding, and eosinophilic inflammation carried out by cytokines released from allergen-specific Th2 cells [12]. Despite common immunologic mechanisms, the immunopathogenesis of allergies is somewhat different according to the disease entity.

Many pharmaceutical therapies have been developed to control allergic diseases. To control histamine effects, antihistamines have been used for a long time, and corticosteroids are widely used for severe allergic conditions. In refractory cases of allergic diseases, cyclosporine A is frequently used, as are other regimens, including alkylating agents [5]. Recently, dupilumab, a monoclonal antibody to the IL-4 receptor α chain, which is the same as the IL-13 receptor α chain, has been actively used for moderate and severe recalcitrant AD [14, 15]. Similarly, omalizumab, an anti-IgE antibody, is used for histamine-mediated diseases, including CU [16]. These are highly specifically targeted pharmaceutical treatments. In dupilumab and omalizumab treatment, remission is induced temporarily and recurs after a period. It is not certain that they are causative treatments. However, from the clinical results of treatment with dupilumab and omalizumab for both recalcitrant AD and CU, it has become clear that the key pathogenesis of AD is mediated by Th2 cells through the IL-4 and IL-13 pathways and that the pathogenesis of CU involves IgE-mediated histamine release.

## Clinical viewpoints of immunopathogenesis of allergic diseases

For the clinical application of new therapeutic modalities, including MSCT, different viewpoints may be needed to understand the clinical aspects of immunopathogenesis.

The first viewpoint to be considered is whether the clinical effects are preventive, causative and/or symptomatic according to the immunopathogenesis [12]. Modulation in the sensitization phase may be preventive. If the action mechanisms permanently affect the effector mechanisms or induce tolerance (desensitization), the treatment modality will be causative. If the therapeutic modality temporarily affects the effector phase and needs to be administered repetitively, it is symptomatic.

The second viewpoint is whether the disease entities are local diseases or systemic diseases [12]. Typical allergic rhinitis (AR) is a disease limited to the nose, and allergic conjunctivitis (AC) is a disease of the eyes. These are organ-specific local diseases and do not necessarily need systemic treatment. In contrast, AD and CU are systemic diseases, showing generalized eczema and urticaria throughout the body. Even in the case of CU, respiratory difficulty is accompanied by airway symptoms and signs resulting from multiorgan involvement. They need systemic treatment. The preparations should be determined from these characteristics. Therapeutic modalities can be applied locally (regionally) or systemically through injection. Systemic administration can be performed for organ-specific effects, as in an animal allergic rhinitis model [17], and in the case of conjunctivitis, the local application of only conditioned media of MSCs has also been used in an animal model [18].

The third clinical viewpoint is whether the immunologic mechanisms of the therapeutic modality are allergen-specific. Classically, allergic diseases depend on the allergenic challenge, as allergen-specific diseases. Allergen-specific treatments are performed, such as desensitization for aeroallergens [19] and tolerance induction for food allergies [4]. Non-allergen-specific treatments such as Histobulin™ (immunoglobulin/histamine complex) and IFN-γ are also possibilities. In the case of CU according to immunopathogenesis, such as autoimmune mechanisms [20], Histobulin™ therapy is also a kind of non-allergen-specific therapy for CU [21], AD [22] and food allergies. Additionally, polydesensitization treatment is conducted when patients show polysensitization to multiple allergens [23]. In the state of polysensitization, IFN-γ and Histobulin™ have polydesensitization effects in a non-allergen-specific manner [21, 22]. Physicians are inevitably pressed to give non-allergen-specific immunotherapy in the case of multiple allergic diseases or if the causative allergens are not identified.

The therapeutic effects of MSCs are also considered from the clinical viewpoint of the immunological roles of MSCs in the immunopathogenesis of AD and CU, which are uniquely representative of disease entities such as in the allergen specificity and the local, systemic, classical or alternative pathogenesis pathways.

## Development of stem cells for mesenchymal stem/stromal cell therapy (MSCT)

**Fig. 2:**
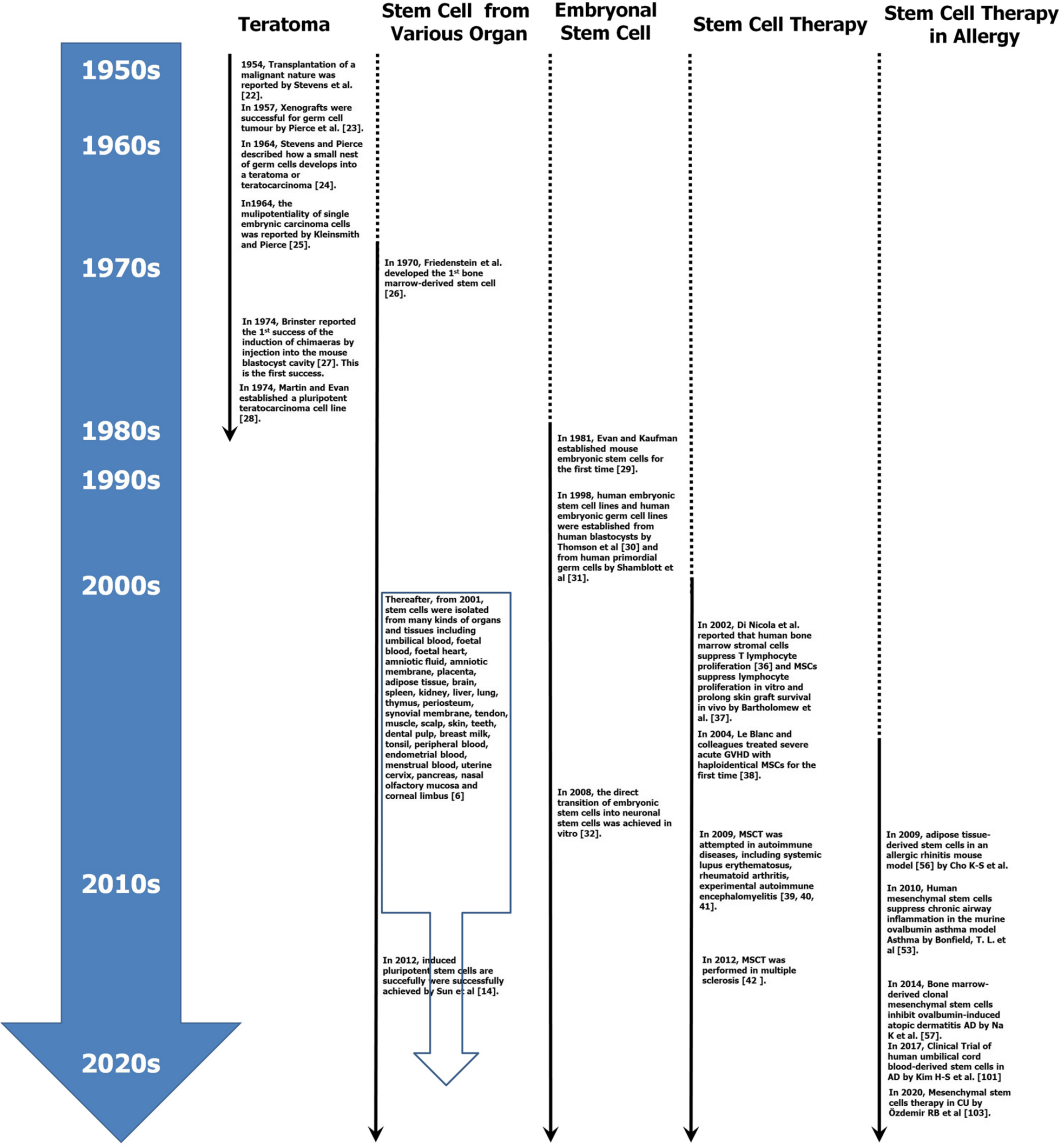
Timeline of landmarks in the development of mesenchymal stem cells and their therapeutic application to allergic diseases

The history of the development of stem cells into MSCT begins with teratoma [24] (Fig. 2). From teratoma, embryonic stem cells were isolated. Later, stem cells were derived from many kinds of organs and tissues. Additionally, induced pluripotential stem cells were also developed.

In 1954, rapid growth by repeated transplantation of a teratoma with a malignant nature was reported by Stevens et al. [25]. In 1957, xenografts were successfully used for germ cell tumours [26].

Stevens and Pierce described how a small nest of germ cells develops into a teratoma or teratocarcinoma [27]. In the same year, the multipotentiality of single embryonic carcinoma cells was reported by Kleinsmith and Pierce [28]. They demonstrated that teratocarcinomas possess a unique type of stem cell, among which a single stem cell once has the capacity to grow indefinitely. Additionally, it was able to differentiate into multiple adult cell types.

Friedenstein et al. developed the first bone marrow-derived stem cell [29].

Brinster reported the first successful induction of chimaeras by injection into the mouse blastocyst cavity [30]. In the same year (1974), Martin and Evan established a pluripotent teratocarcinoma cell line. The cells proliferated indefinitely and produced teratocarcinoma upon subcutaneous injection with differentiation into multiple adult cell types [31].

Research on teratocarcinoma with a focus on stem cells has mostly dwindled over the last 10 years, and teratocarcinomas and embryonal carcinoma cells are not used today. However, the data from this research are critical for the development of embryonic stem cells.

Evan and Kaufman established mouse embryonic stem cells for the first time [32]. In 1998, human embryonic stem cell lines and human embryonic germ cell lines were established from human blastocysts by Thomson et al. [33] and from human primordial germ cells by Shamblott et al. [34].

Thereafter, from 2001 to 2016, stem cells were isolated from many kinds of organs and tissues, including umbilical blood, foetal blood, foetal heart, amniotic fluid, amniotic membrane, placenta, adipose tissue, brain, spleen, kidney, liver, lung, thymus, periosteum, synovial membrane, tendon, muscle, scalp, skin, teeth, dental pulp, breast milk, tonsil, peripheral blood, endometrial blood, menstrual blood, uterine cervix, pancreas, nasal olfactory mucosa and corneal limbus [6], in addition to the isolation of the 1^st^ bone marrow-derived stem cells in 1970 [29].

In 2008, the direct differentiation of embryonic stem cells into neuronal stem cells was achieved in vitro [35]. Induced pluripotent stem cells were successfully developed by Sun et al. in 2012. [17].

## Mesenchymal stem cell therapy (MSCT)

First, the features of MSCs make them a potential therapeutic tool for inflammatory diseases [36]. MSCs have tissue repair potential through their self-renewal and differentiation abilities and are increasingly considered regulators of immune responses [37, 38].

### Anti-inflammatory effects

The first clinical application of MSCs was in the treatment of severe acute graft-versus-host disease (GVHD). Di Nicola et al. reported that human bone marrow stromal cells suppress T lymphocyte proliferation [39], and Bartholomew et al. found that MSCs suppress lymphocyte proliferation in vitro and prolong skin graft survival in vivo [40]. Le Blanc and colleagues treated severe acute GVHD with haploidentical MSCs for the first time [41].

In 2009, MSCT was attempted for autoimmune diseases, including systemic lupus erythematosus and rheumatoid arthritis, in 2009, with beneficial outcomes [42, 43]. In the same year, it was reported that the effects of MSCs in experimental autoimmune encephalomyelitis are mediated by transforming growth factor-β and interleukin (IL)-6 [44], and MSCT was performed in multiple sclerosis in 2012 [45].

### Tissue repair and regeneration

MSCs can modulate the tissue repair process by differentiating into various types of cells [46]. MSCs are considered and tried for the regeneration of damaged tissue in several disease states, such as cardiovascular disorders [47], liver damage [48], kidney injury [49], bone diseases [50] and neurological defects [51].

The MSCs that have been investigated have come mostly from the umbilical cord, bone marrow and adipose tissue. Recently, stromal vascular fraction (SVF)-based cellular therapies have rapidly advanced and been applied in various clinical settings, including scars [52, 53], hemifacial atrophy [54], breast reconstruction [55], wound healing and cancer therapy [56], hair regrowth [57], breast augmentation [58] and also in vitro applications [59]. The SVF portion contains stromal vascular fraction cells (SVFCs) and adipose stem cells (ASCs) [60]. SVF may be easily obtained from human adipose tissue and is a rich source of ASCs [61]. Early on, SVFCs and the related ASCs were used in regenerative plastic surgery for autologous implantation for several years. Thereafter, allogenic implantation was evaluated for the treatment of perianal fistulas, diabetic foot ulcers, knee osteoarthritis, acute respiratory distress syndrome, refractory rheumatoid arthritis, paediatric disease, faecal incontinence, ischaemic heart disease, autoimmune encephalomyelitis, lateral epicondylitis and soft tissue defects with effectiveness and safety. Decellularized extracellular matrix (ECM) was introduced as a biological scaffold for human tissue engineering [62–64]. Allogenic SVF transplants were accomplished with decellularized ECM from a donor and re-cellularized by ASCs of the recipient.

For several years, ASCs have been routinely used in regenerative surgery, and recently, their potential pro-oncogenic or anti-oncogenic role was considered and reviewed [65]. ASCs have proven to favour tumour progression but are regarded as a potentially suitable vehicle for the delivery of new anti-cancer molecules into the tumour microenvironment because of their high secretory activity that preferentially targets them to tumours.

Mesenchymal cells within the SVF of subcutaneous adipose tissue display multilineage developmental plasticity in vitro and in vivo [66]. Zuk et al. reported multilineage cells from human adipose tissue as a source of multipotent stem cells [67, 68]. Halvorsen reported ECM mineralization and osteoblast gene expression by human ASCs [69] and the chondrogenic potential of ASCs was revealed in vitro and in vivo by Erickson et al. [70]. Moreover, neurogenic differentiation of murine and human ASCs was reported by Safford [71]. Katz et al. [66] analysed the cell surface of and transcriptionally characterized human adipose-derived adherent stromal cells.

Proponents of platelet-rich plasma (PRP) technology suggest that its benefits include an increase in hard- and soft-tissue wound healing and a decrease in postoperative infection, pain and blood loss [72]. In 2002, the clinical use of platelet-rich plasma for a wide variety of applications was reported, most prevalently for problematic wounds, maxillofacial defects and spine defects [73, 74]. In 2002, a paper was published on the dose–response relationship between platelet concentration and the proliferation of human adult MSCs, the proliferation of fibroblasts and the production of type I collagen in vitro [75]. This suggests that the application of autogenous platelet-rich plasma can enhance wound healing, as has been demonstrated in controlled animal studies for both soft and hard tissues [76, 77].

In 2006, platelet-rich plasma was reported to stimulate adipose tissue regeneration in controlled animal studies for soft and hard tissues [74]. In 2013, it was suggested that growth factors, including vascular endothelial growth factor, fibroblast growth factor and platelet-derived growth factor-BB, which are present in the PRP, play a role in improving tissue healing [78].

The results of clinical application of PRP in 2014 were dramatic in patients with scars on the face [79]. The report drew four fundamental conclusions: (1) PRP sustains an optimal microenvironment that leads to correct architectural adipocyte distribution, better cell-to-cell interactions, adipose tissue growth and differentiation from ASCs; (2) PRP facilitates the delivery of proper nutrient and oxygen levels to grafted cells b inducing early development of the neoangiogenic microcapillary network; (3) SVF boosts the neoangiogenic vascularization and fibrogenic activity of fibroblasts, which favour adipose tissue survival and three-dimensional organization; and (4) SVF and PRP improve fat graft maintenance in patients who underwent regenerative surgery.

Biomaterials, cells and growth factors are needed to design a regenerative plastic surgery approach in the treatment of organ and tissue defects, and growth factors have also been a focus in SVF with PRP therapy [80, 81]. 3D collagen scaffold culture in association with platelet-derived growth factors and insulin favours the chondrogenic and osteogenic differentiation of ASCs. These results suggest new translational applications in regenerative medicine for the management of osteochondral defects [80]. Additionally, growth factors contained in PRP, including angiogenic factors and osteogenic factors, have been used to promote tissue formation in soft tissue defects, periodontal defects, oral surgery, maxillofacial surgery, aesthetic plastic surgery, spinal fusion and heart bypass surgery [81].

In 2017, stem cells from human hair follicles were isolated for the first time for immediate autologous clinical use in androgenetic alopecia and hair loss [82], and autologous regenerative stem cell therapy has been done for alopecia [83]. Additionally, the introduction of PRP improved hair regrowth by autologous human follicle MSC therapy in androgenetic alopecia [84]. The effects of autologous non-activated PRP and activated PRP in wound healing and hair regrowth were evaluated [85], and in 2021, the Academy of International Regenerative Medicine and Surgery Societies (AIRMESS) recommendations on the use of platelet-rich plasma (PRP) and autologous stem cell-based therapy (ASC-BT) in androgenetic alopecia and wound healing were released [86].

SVFCs have been used for soft tissue defects in radiotherapy-based tissue damage after mastectomy, breast augmentation, calvarial defects, Crohn’s fistulas and damaged skeletal muscle [87, 88]. Several studies have been performed to improve the results of fat grafts concerning the maintenance of fat volume and prevention of reabsorption. PRP was introduced in this field, and breast reconstruction with autologous fat grafts mixed with PRP was effectively conducted in 2014 [89]. In 2015, to enhance the effectiveness of fat grafts, enhanced SVF fat grafting was tried [90]. The nanofat procedure was proposed to improve tissue repair by the stem cells contained in the SVF of nanofat in 2017 [91], and engineered fat grafts enhanced with SVFCs were tried, resulting in increased graft survival and function in patients who underwent breast reconstruction and oncoplastic surgery [88]. With the increase in autologous therapies using adipose-derived SVF and adult ASCs, the methods for preparation are continually advancing, such as enzymatic digestion and mechanical centrifugation [92].

Currently, the application of SVF and ACS in regenerative medicine for soft tissue defects targets the local defect area. Allergic diseases, including AD and CU, are also allergic inflammation, and tissue repair and regeneration are important points in the recovery from allergic inflammation. In the case of local application at the lesion site, SVF and ASC may be effective. However, the basic concept of this review of allergic diseases is the systemic roles of MSCs. Moreover, PRP contains many bioactive materials, including growth factors. The application of PRP should be carefully considered in terms of the bioactive materials in PRP as well as the roles of platelets, whether they are beneficial or harmful, because the immunopathogeneses of AD and CU are different from general inflammation.

### Mesenchymal stem/stromal cell therapy (MSCT) in severe acute respiratory syndrome coronavirus 2 (SARS-CoV-2)

MSCs have been tested in viral infections, such as cytomegaloviral infection [93] and acute lung injury caused by influenza virus [94], using different tissue-derived MSCs. MSCT has been recommended for the prophylaxis of infections in patients undergoing high-dose chemotherapy [95].

In 2019, severe acute respiratory syndrome coronavirus-2 (SARS-CoV-2) caused a pandemic spurring an unprecedented global crisis. In SARS-CoV-2 infection, acute respiratory distress syndrome (ARDS) is the major problem, and the cytokine storm can be fatal [96]. MSCT for ARDS has been tried recently, with some success. [97]. ASCs were suggested as a new regenerative immediate therapy combating SARS-CoV-2-induced pneumonia [98].

Cytokine storm is one of the major problems of fatality in SARS-CoV-2 infection [96] and sepsis [99]. In the cytokine storm, pro-inflammatory and anti-inflammatory pathways are upregulated with simultaneous immunosuppression, which results in a state of immunoparalysis [100]. The role of IL-6 was the focus [101], and cytokine storm with rapidly elevated IL-6 was suggested as an indicator for sudden death in patients with critical SARS-CoV 2 infection [102]. Regarding the anti-inflammatory and immunomodulatory activities of SVFCs and ASCs, as described below, they are considered a potential cellular therapy in SARS-CoV-2 infection [103, 104]. Currently, there are no approved MSC-based approaches for the prevention or treatment of SARS-CoV 2 infection, but many clinical trials are ongoing [105].

### Immunomodulatory effects

Di Nocola et al. reported that human bone marrow stromal cells (BMSCs) suppress T lymphocyte proliferation [39]. In their investigation of the mechanisms underlying BMSC-mediated T cell suppression, although the addition of neutralizing monoclonal antibodies anti–rhIL-6 and anti–rhIL-10 failed to restore T-cell proliferation suppressed by BMSCs, the addition of monoclonal antibodies neutralizing either rhTGF-β or rhHGF increased BMSC-suppressed T-lymphocyte proliferation. The production of TGF-β and HGF from MSCs was demonstrated for the first time.

At first, the main focus of the immunomodulatory effects of MSCs was their immunosuppression. Immunosuppression was shown to be mediated by the production of IL-10 in 2006 [106], nitric oxide in 2007 [107], prostaglandin E2 (PGE2) in 2005 [108] and indoleamine 2,3-dioxygenase (IDO) in 2004 [109], in addition to TGF-β and HGF. In a study of MSC effects in experimental autoimmune encephalomyelitis, the production of IL-6 and TGF-β was also demonstrated in 2009 [36]. The production of TGF-β and IL-6 affected the Treg and Th17 cell balance. On the background of these results, MSCs are increasingly considered regulators of immune responses [38]. Thereafter, a series of studies was published to investigate the therapeutic efficacies and relevant mechanisms of the immunomodulatory action of MSCs.

## Anti-allergic effects of immunomodulatory mesenchymal stem/stromal cells (MSTs) in allergic diseases

Initially, MSCT in allergic disease was approached from the perspective that allergic diseases are specific anti-inflammatory conditions and MSCs may exert consistent anti-inflammatory effects to have therapeutic efficacy against different disease-specific inflammatory statuses. A number of recent studies have demonstrated that MSCs can also alleviate allergic immune disorders, such as asthma [110, 111], allergic rhinitis [112] and AD [113]. Many immunologic anti-allergic mechanisms have been revealed through MSC therapy experiments in animal asthma models. Through research on allergic diseases in animal models and clinical trials, anti-allergic mechanisms were deduced, and clinical effects might be predicted. Currently, for the clinical application of MSCs in AD and CU, a systemic approach to the critical immunomodulatory properties of MSCs and critical analysis of their therapeutic potential is absolutely necessary. Below, we highlight the immunologic roles of MSCs with the matching immunopathogenesis of allergic diseases from a clinical viewpoint to understand the nature of MSCs for the successful MSCT in allergic diseases.

### Anti-allergic mechanisms of MSCs in asthma

Asthma was the first allergic disease in which MSCs were applied in animal models. Bonfield et al. reported that intravenous injection of MSCs suppressed chronic airway inflammation in a murine ovalbumin asthma model [110]. Several important results were obtained in this study. Eosinophils, IL-5, IL-13 and IFN-γ in bronchoalveolar lavage fluid (BALF) were decreased significantly. Moreover, systemic IgE was decreased. The potential usefulness of hMSCs in severe uncontrolled chronic asthma was suggested.

This study conveyed several important messages on the immunologic characteristics of MSCs. MSCs exert local anti-allergic effects by reducing IL-5, IL-13 and eosinophils despite systemic administration of MSCs. MSCs have systemic effects by reducing total IgE. The reduction in IFN-γ along with IL-5 and IL-13 is the most desired result for the treatment of AD considering the immunopathogenesis of acute and chronic AD. Thereafter, MSCs were reported to reduce lung inflammation and tissue remodelling in allergic asthma in an animal model [111, 114, 115].

Human-induced pluripotent stem cell-derived MSCs (hiPSC-MSCs) and bone marrow-derived MSCs were administered systemically by IV injection in a murine asthma model [111]. Locally, there was a significant decrease in IL-5 and IL-13 in the BALF and IL-4 in the NALF, and systemically, hiPSC-MSCs decreased the circulating levels of OVA-specific IgE and IgG1.

Nemeth reported that Th2-related cytokines activated the signal transducer and activator of transcription 6 (STAT6) pathway in BM-MSCs, which elevated their production of TGF-β, which in turn contributed to the attenuation of asthma in mice [116].

Goodwin et al. reported that the inhibition of Th2-mediated inflammation may be related to the enhancement of Th1 cell generation. They silenced the IFN-γ gene in mice with asthma, and systemic administration of BM-MSCs failed to suppress eosinophils and Th2-related cytokines. This result indicates that MSCs exert their immunomodulatory effects through an IFN-γ-dependent process [117].

In 2013, BM-MSC infusion upregulated IL-12 levels and downregulated IL-4, IL-13, OVA-specific IgE, OVA-specific IgG1 and OVA-specific IgG2a levels in a mouse asthma model [118]. Dental follicle MSCs inhibited the proliferation of CD4+ T cells and reduced effector and effector memory CD4+ T cell numbers in a mouse asthma model [119].

Intravenous transplantation of human placental MSCs elevated Tregs and serum IL-10 in the peripheral blood of rats with asthma [120]. This suppression was mediated by TGF-β, and the increase in Tregs was also stable over time [116].

Systemic administration of adipose tissue-derived MSCs (AD-MSCs) restored the number of Tregs and rescued the impairment of IL-10, Foxp3, and IL-17 levels [121]. The increase in Tregs by MSCs was related to haeme oxygenase-1 (HO-1) activity [122]. Human umbilical cord blood (hUCB)-MSCs increased the IL-10 production of Tregs in a mouse asthma model [123]. As seen from these effects, MSCs shift the cytokines towards a new Th1/Th2 balance and modulate T cell proliferation.

### Anti-allergic mechanisms of MSCs in allergic rhinitis

In 2009, systemic administration of AD-MSCs in a murine allergic rhinitis (AR) model was the first trial of MSCT in AR [112]. MSCs were localized to the nasal mucosa by systemic administration, and the signs of allergic rhinitis were improved. Eosinophil infiltration was decreased in the nasal mucosa by shifting to a Th1-type response from a Th2-type immune reaction to allergens. Serum allergen-specific IgE was decreased and IgG2a was increased. Of the cytokines produced by splenocytes, IL-4 and IL-5 decreased and IFN-γ increased, similar to the results in animal asthma models.

In 2012, in vitro, human iPSC-MSCs regulated T cell phenotypes towards the suppressive Th2 phenotype by inducing Treg expansion, which was associated with PGE2 expression and cell–cell contact in human allergic rhinitis [17]. Tregs were induced allergen-specifically, which might lead to tolerance to the allergens. In 2018, enhancement of Tregs by iPSC-MSCs was reported to occur via NF-kB signalling in vitro [124].

In 2015, tonsil-derived MSCs (T-MSCs) significantly attenuated allergic symptoms in an AR mouse model [125]. T-MSCs inhibited Th2-associated mediators and IgE production in B cells, and the levels of IL-25, IL-33 and eosinophil chemokines, including eotaxin-1 (CCL11) and eotaxin2 (CCL24), were suppressed in the nasal mucosa.

Systemic administration of MSCs enhanced the Th-1 immune response by upregulating serum IFN-γ levels, while it inhibited the Th-2 immune response by downregulating serum IL-4, IL-5 and IL-10 levels in an AR mouse model [126]. Additionally, serum total IgE decreased and IgG2a increased. Similar effects and mechanisms of BM-MSCs were confirmed [43, 44].

Systemic administration of hUCB-MSCs inhibited histamine secretion in rats with AR [127]. Serum IgE, IL-4, and IL-17 levels decreased, and serum IFN-γ levels increased, as in other reports. Moreover, TNF-α and serum histamine levels were decreased in this study.

### Anti-allergic mechanisms of MSCs in allergic conjunctivitis

In 2015, the local instillation of conditioned media (CM) from TNF-α-stimulated BM-MSCs attenuated the clinical signs of experimental allergic conjunctivitis (AC) [18]. CM decreased mast cell activity and IgE levels in B cells and alleviated the vascular hyperpermeability induced by histamine in vitro. In vivo, CM suppressed the secretion of IgE and histamine, the recruitment and activity of MCs, and the hyperpermeability of vessels in the conjunctiva. The anti-allergic effect of CM in AC might be mediated by COX2.

### Anti-allergic mechanisms of MSCs in allergic contact dermatitis

MSCs that were systemically administered by intravenous injection preferentially migrated into the draining lymph node and produced NO to promote T cell apoptosis, thereby improving allergic contact dermatitis (ACD) [128]. IFN-γ, but not IL-10, expression was decreased, which resulted in ACD cure in a self-limiting course [129].

MSCs suppress TNF-α and IFN-γ secretion by CD8+ T cells by silencing stannoicalcin-2 (STC2) [130]. Systemic administration of human gingiva-derived MSCs (GMSCs) accentuates an increase in the local Treg numbers and IL-10 expression in the draining lymph node and allergic ear tissue [131]. Regional injection of GMSCs was more effective than systemic infusion during the late phase of CHS. These GMSC effects were achieved through prostaglandin E receptor-3 (EP3).

## Immunomodulatory roles of mesenchymal stem/stromal cells in the immunopathogenesis of atopic dermatitis and chronic urticaria: predicted and proven immunological mechanisms

Anti-inflammatory effects are beneficial for allergic diseases, especially asthma and AD. However, the anti-allergic effects of MSCs may be the more important and specific concept for the application of MSCT to AD and CU. The anti-allergic effects of MSCs from previous studies on allergic diseases before MSCT in AD and CU are described below, matching their effects to the immunopathogenesis of AD and CU.

### Atopic dermatitis

The classic pathway of the immunopathogenesis of AD schematically begins with sensitization to allergens and is followed by effector function under rechallenge with allergens [12]. In the sensitization phase, allergen-specific IgE and allergen-specific Th2 cells are produced in response to a Th1/Th2 imbalance. In the effector phase, allergen binding to allergen-specific IgE and allergen-specific Th2 cell activation and histamine release from mast cells through FcRε with rechallenge by sensitized allergen, Th2 cytokine release and eosinophilic recruitment result in eczema and eosinophilic inflammation. Acquisition of allergen-specific tolerance should also be considered. On the basis of the schematic immunopathogenesis of AD, the effects are classified as preventive effects, causative treatment and symptomatic treatment.

In the sensitization phase, systemic administration of MSCs restores the Th1/Th2 imbalance by reducing Th2 cytokines and elevating IFN-γ levels [12]. These effects possibly reduce or prevent allergen sensitization, resulting in the reduction or prevention of allergen-specific IgE production and allergen-specific Th2 production. This may be the preventive effects of allergies by MSCs.

In the effector phase of AD, IgE-mediated reactions and Th2 cell-mediated reactions should be considered. In the IgE-mediated pathway, MSCs reduce allergen-specific IgE [12]. This may be a causative treatment. MSCs inhibit mast cell degranulation and consequently inhibit histamine release, which occurs by binding allergens to allergen-specific IgE via FcRε on mast cells [132]. TGF-β1, which is produced by MSCs, inhibits the FceRI expression of mast cells, a critical component for IgE-mediated mast cell degranulation [116, 133]. This may therefore be a symptomatic treatment. Concerning the Th2-cell-mediated pathway, the production of allergen-specific Th2 cytokines, including IL-4, IL-5 and IL-13, is decreased by MSCs. Additionally, systemic and lesional eosinophils are decreased by MSCs. These factors lead to symptomatic improvement (symptomatic treatment).

**Fig. 3:**
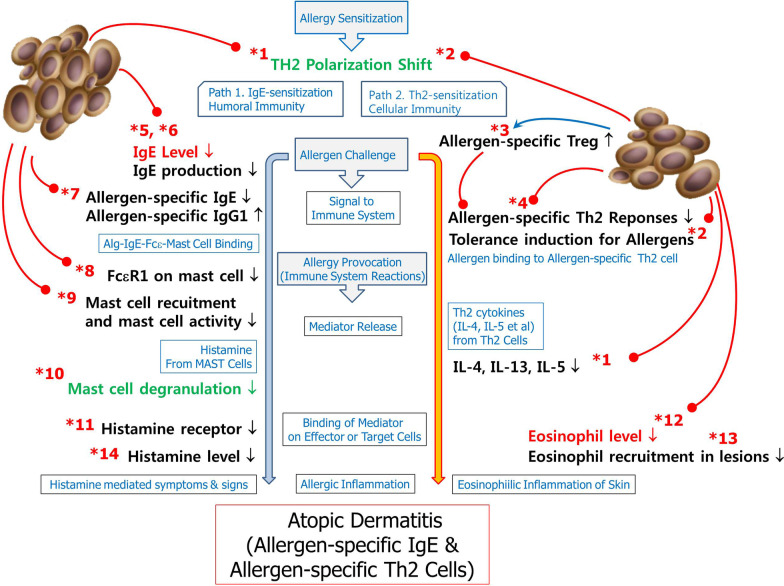
Possible immunologic roles of MSCs by which therapeutic effects were expected and proven in AD. The numbers marked with asterisks (*) represent the immunologic mechanisms of MSCs in Table 1. Red arrows represent negative regulation, and blue arrows represent positive regulation. Blue text represents the flow of immunopathogenesis. The immunologic roles of MSCs are presented in black text for animal models of allergic conditions other than AD, green text for animal models of AD, and red text for human clinical trials

For tolerance induction, IFN-γ, IL-10 and TGF-β play roles. IFN-γ has effects on allergen-specific tolerance induction for food allergies in AD and drug allergies [19]. Allergen-specific regulatory B cells, including IL-10-producing CD5+ B cells (Br1s) [134], Foxp3-expressing CD5+ B cells (Bregs) [135] and TGF-β-producing CD5+ B cells (Br3s) [136], are involved in tolerance mechanisms in AD. IL-10 induces tolerance through allergen-specific anergy [137]. MSCs increase IL-10, and the acquisition of tolerance to allergens through allergen-specific anergy may be induced. Regulatory T cells (Tregs) are involved in immunologic tolerance [138]. MSCs increase allergen-specific regulatory T cells [44], and tolerance to allergens may be induced through allergen-specific Tregs. Tolerance induction is a causative treatment. From the results of previous studies, MSCT is sufficiently effective against AD, with preventive effects, namely, allergen-specific tolerance induction as a causative treatment and symptomatic relief as a symptomatic treatment. The predicted immunological roles from the immunological mechanisms of MSCs that were discovered by studying allergic diseases other than AD and by studying the immunologic mechanisms in AD are listed briefly (Table 2, Fig. 3).

**Table 2:** The immunologic mechanisms of MSCs in allergic diseases

Immunological mechanisms	Applicable disease			Data resources			Phase
	No	AD	CU	Animal models of AS*,* AR, AC and ACD	Animal AD models	Human trial	
*T cell*							
Thl/Th2 balancing by modulating Th2 polarization				Thl cytokines			Sensitization/effector
				IL-12: mouse AS (118)	Thl cytokines		
				IFN-γ: mouse AS (116)	IFN-γ mouse (150)		
	*l	O		Th2 cytokines	Th2 cytokines		
				IL-4: mouse AS (110, 118), rat AR (156)	IL-4: mouse (112, 150)		
				IL-5: mouse AS (113), mouse AR (125)	IL-5: mouse (150)		
				IL-13: mouse AS (110, 118, 122), mouse AR (124)			
Production or induction of regulatory cytokines related with tolerance/desensitization (TGF-β, IL-10, IFN-γ)	*2	O		TGF-β: mouse AS (115), rat AS (119)	TGF-β: mouse (151)		Sensitization/effector
				IL-10: mouse AS (119, 122)			
				IFN-γ: mouse AS (116)			
Increase allergen-specific regulatory T cell (Treg) response	*3	O		rat AS (44),mouse AS (121), mouse AR (17), mouse ACD (129)			Sensitization/effector
Reducing allergen-specific Th2 response	*4	O					
*B cell*							
Decrease of systemic lgE	*5	O		mouse AS (109), mouse AR (44, 124, 125), rat AR (126), mouse AC (18)		hAD (156)	Effector
Decrease of IgE production	*6	O	O	mouse AS (109), mouse AR (44, 124, 125), rat AR (126), mouse AC (18)			Effector
Reducing allergen-specific IgE and increasing allergen-specific IgGl	*7	O		Mouse AS (110, 118), mouse AR (111, 124)	mouse (112)		Effector
*Mast cell*							
Decrease of IgE binding to mast cell by reducing FccRl	*8						
Decrease of mast cell recruitment and activity	*9	O	O	mouse AC (18)			Effector

*AD* atopic dermatitis, *hAD* human atopic dermatitis, *CU* chronic urticarial, *AS* asthma, *AR* allergic rhinitis, *AC* allergic conjunctivitis, *ACD* allergic contact dermatitis

### Chronic urticaria

CU is a disease in which urticaria repetitively develops for more than 6 weeks [8]. The prevalence of CU is 0.02–5% and tends to increase each year. CU is classified as chronic spontaneous urticaria (CSU) and chronic inducible urticaria (CIU). The main event in the CSU pathway is IgE-mediated mast cell degranulation with the release of mediators, including histamine. Up to 30–50% of idiopathic cases may be autoimmune or related to mast cells and basophil abnormalities. Autoantibodies to the high-affinity receptor for IgE (FcRεI), which specifically bind to the α chain, may be pathogenic. The gold standard for detecting clinically relevant autoantibodies to FcRεI is the functional in vitro donor basophil histamine release assay. The final common pathway of CU is the release of histamine and other proinflammatory factors following the degranulation of mast cells. CU by autoimmune mechanisms is an alternative pathway in the pathogenesis of allergies. CU developed without allergens and allergen-specific IgE. Th2 levels in CSU were reported to be elevated [139–141]. Tregs did not show consistent results and were significantly higher [142], lower [143], or similar [139]. The unresponsiveness of omalizumab in the CU was reported to be related to the immunopathogenesis of CU [16]. Interestingly, MSCs are effective against autoimmune disease [42].

MSCs inhibited the FceRI expression of mast cells through TGF-β1 action, which is produced by MSCs [116, 133]. IL-4-induced FcεR1 expression on mast cells was decreased by TGF-β, which is produced by IL-4 via STAT6 signalling of MSCs [132]. FcεRI was a critical component for IgE-mediated mast cell degranulation [133].

Conditioned media from MSCs decreased mast cell activity and recruitment and induced the release of histamine [18]. MSCs suppress the degranulation of mast cells in vivo and in vitro [132]. BM-MSCs suppress mast cell functions via COX2-dependent mechanisms [144]. Multiple PGE2 receptors (EP receptors) on mast cells differentially regulate the response of MCs by PGE2 stimulation [145]. MSCs suppress mast cell degranulation by producing PGE2 via NOD2-RIP2-COX-2 signalling [132]. As expected from these experimental data, MSCs decreased the blood level of histamine in an animal model [127].

MSCs reduced reactive oxygen species (ROS), which trigger mast cell activation through both FcεRI and histamine H4 receptor (H4R)-dependent pathways [146–148]. MSCs reduced H4R expression through the inhibition of nuclear factor-kappa B (NFκB), which binds to the H4R promoter region and drives H4R upregulation and activation [149].

**Fig. 4:**
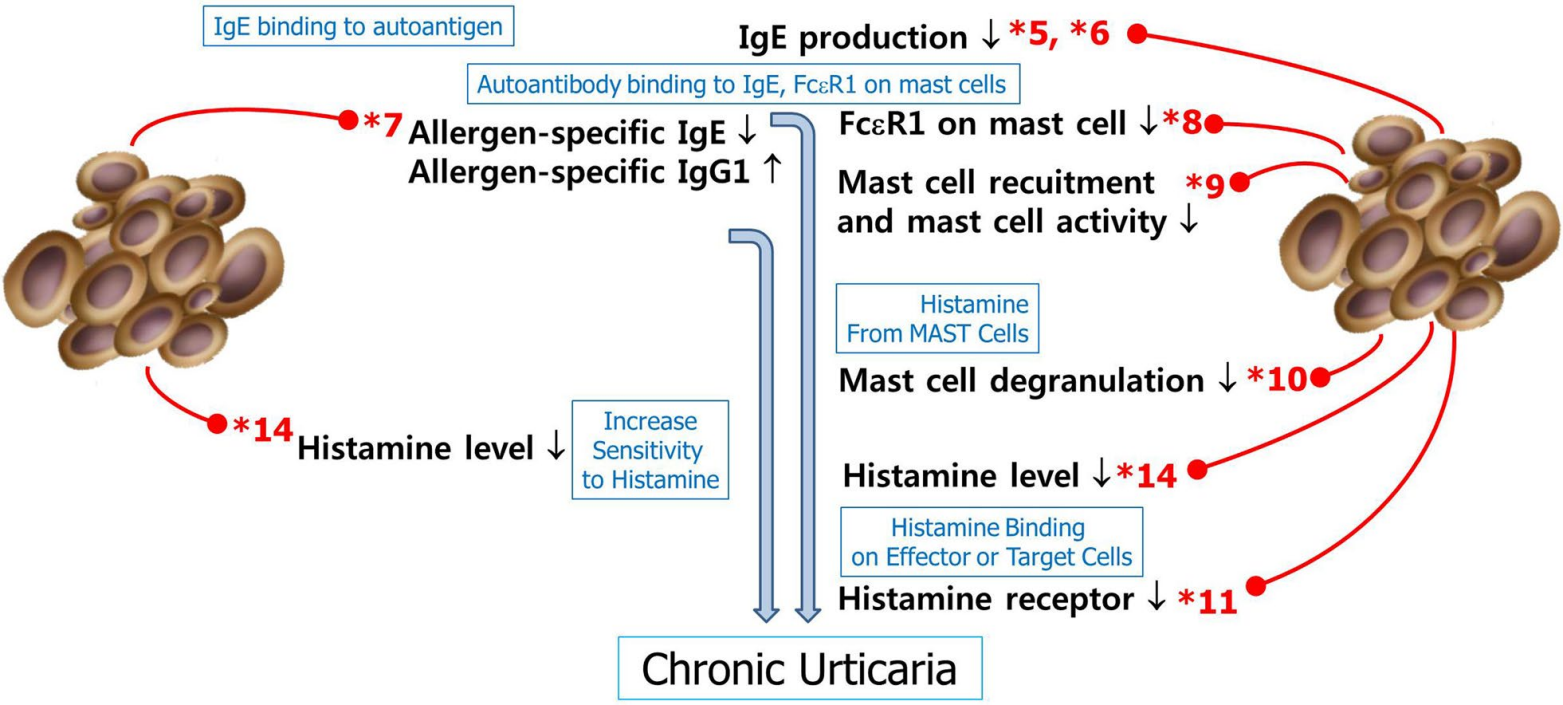
Possible immunologic roles of MSCs by which therapeutic effects were expected to occur in animal models of allergic diseases other than CU. Blue text represents the flow of immunopathogenesis of CU, and black text represents the role of MSCs in allergic diseases

Through these immunologic mechanisms, MSCs are expected to treat all aspects of the pathogenesis of CU and thus seem to be highly suitable for the treatment of CU (Table 2, Fig. 4).

## Mesenchymal stem/stromal cell therapy (MSCT) in AD and CU

### Mesenchymal stem/stromal cell therapy (MSCT) in AD

#### Animal studies

In 2010, autogenous adipose-derived MSCs (AD-MSCs) were applied for the treatment of canine AD [150]. However, the AD-MSCs did not significantly reduce the clinical signs of canine AD.

In 2014, the first successful MSCT for AD was achieved in a mouse AD model [113]. Syngeneic and allogeneic grafts in BM-MSCs were performed. Intravenous administration of murine BM-MSCs suppressed cell infiltration in skin tissue and reduced IgE levels in serum. IL-4 expression in lymph nodes and cutaneous tissues was inhibited. MSCs inhibited B cell differentiation, T cell activities and cytokine production, which resulted in a beneficial effect.

Mechanistic studies showed that their immunosuppressive effects came from the significant inhibition of T cell proliferation by syngeneic and allogeneic MSCs. Both T-bet and GATA-3, which are transcription factors for the IFN-γ and IL-4 genes, respectively, were suppressed by MSCs. IgE production in culture media was significantly suppressed by MSCs, and this suppression was cell–cell contact dependent. MSCs inhibit B-cell proliferation and IgE production via cell–cell contact.

In an in vivo study, MSCs migrated to skin lesions and draining lymph nodes in an AD mouse model. To exclude an allograft rejection response in this AD mouse model, both allogeneic and syngeneic MSCs were used. Cell infiltration in the skin was decreased in mice treated with both allogeneic and syngeneic MSCs. The severity score of skin lesions and the thickness of the epidermis and dermis were significantly decreased by MSCs.

Systemically, mast cells, T cells and eosinophils were also significantly decreased by MSCs. OVA-specific IgE levels were significantly decreased, and the total level of IgG2a was significantly increased, without a significant change in OVA-specific IgG2a.

Local immunological effects of MSCT were that IL-4 mRNA expression in lymph nodes and skin was significantly decreased by MSCs, consistent with the in vitro results. The expression of IFN-γ, IL-10 and TGF-β in lymph nodes was not changed by MSCs. The expression of Foxp3, which is a marker for regulatory T cells, was also not affected by MSCs.

In 2015, a study of MSCT in a murine AD models [132] found that systemic subcutaneous administration of nucleotide-binding oligomerization domain 2 (NOD2)-activated hUCB-MSCs as a xenograft had a powerful therapeutic benefit in AD and inhibited the infiltration and degranulation of mast cells via increased production of PGE2 and TGF-β1. MDP-stimulated hUCB-MSCs showed robust protective effects against Df-induced AD in mice (preventive effects). These results mean that allergen sensitization may be prevented by MSCT. Subcutaneous administration of MDP-MSCs allows them to protect against AD symptoms by inhibiting the infiltration and degranulation of locally acting mast cells, and MDP-MSCs exert therapeutic effects against developed AD.

MSC injection significantly ameliorated the symptoms of induced AD by decreasing the clinical severity and epidermal hyperplasia. MDP-MSCs prevented the degranulation of mast cells through the activation of NOD2 signalling to COX-2 in response to MDP. In vitro, MSCs exerted an inhibitory effect on mast cell degranulation. MDP-MSCs suppressed mast cell degranulation by producing PGE2 via NOD2-RIP2-COX-2 signalling. hUCB-MSCs efficiently inhibited mast cell degranulation independently of cell-to-cell contact through a higher production of PGE2.

In 2017, human adipose tissue-derived MSC (hAT-MSC) therapy was performed in a murine AD model [151]. hAT-MSCs were systemically administered by intravenous injection as xenografts.

The human AD-MSCs not only inhibited the function of MCs but also clearly suppressed the proliferation and maturation of B cells via cyclooxygenase (COX)-2 signalling in an experimental AD animal model. Interestingly, intravenous administration of high-dose hAT-MSCs significantly reduced the clinical severity of AD in mice. The serum level of IgE was decreased significantly by hAT-MSC therapy in a dose-dependent manner. Additionally, epidermal hyperplasia and lymphocyte infiltration were attenuated, and the number of degranulated MCs was reduced significantly.

Intravenously injected hAT-MSCs were mostly distributed in the lungs and hearts of mice and excreted within 2 weeks; at weeks 2 and 4, hATMSCs were not detected in any of the evaluated organs of mice. All mice that were administered hAT-MSCs survived until sacrifice and did not show any adverse effects. The suppression of the proliferation and maturation of B lymphocytes by hAT-MSC therapy occurred via COX-2 signalling. Inhibition of mast cell degranulation by hAT-MSCs occurred through the concerted action of TGF-β1 and COX-2 signalling.

Skin-derived MSCs (S-MSCs) from the lesional skin of AD patients secrete more Th1/Th17 cytokines, whereas the levels of Th2 factors are lower than those of MSCs derived from the skin (S-MSCs) of healthy people [152]. This finding suggests that MSCs modulate the Th1/Th17 balance in AD patients. Additionally, it has been demonstrated that human AD-MSC-derived exosomes reduce pathological symptoms such as the clinical severity and number of mast cells in the dermal tissue in AD mouse models [153].

In 2018, canine adipose MSCs (cAD-MSCs) were tested in dogs with refractory AD, and systemic administration of allogeneic cAD-MSCs by intravenous injection appeared to be effective, producing positive outcomes in terms of the remission of clinical signs of AD refractory to conventional medications for at least 6 months, with no adverse events [154].

The priming of MSCs was attempted to enhance their therapeutic effectiveness. In 2018, the therapeutic effects of human MSCs were enhanced by transduction with superoxide dismutase 3 in a murine AD-like skin inflammation model [146]. In 2019, hUCB-MSCs were subcutaneously infused after priming with mast cell granules in murine AD models, and the therapeutic effect was improved [155]. In the same year (2019), subcutaneous administration of Poly I:C- or IFN-γ-primed Wharton’s jelly-derived MSCs (WJ-MSCs) improved the therapeutic effects in a murine AD model [156]

#### Human studies and ongoing clinical trials

In 2017, the first clinical trial of MSCT was conducted in AD as a phase I/IIa trial [157] (Table 3). Human umbilical code blood-derived MSCs (hUCB-MSCs) were administered by subcutaneous injection with 2 × 10^7^ as a lower dose and 5 × 10^7^ as a higher dose for moderate to severe AD. A total of 37 patients were involved. Treatment was performed every 2 weeks for 12 weeks.

**Table 3:** Clinical trials of mesenchymal stem cell therapy in human allergic disease

	Graft type	MSC source	Study type	Route	Dose	*N*	Frequency	Interval	Response	Side effects
AD [157]	Allograft	Umbilical cord blood	Phase 1	SQ	2.5 × 10^7^ (Low dose)	3	Single dose		Favourable	No severe side effects
					5 × 10^7^ (High dose)	3				
			Phase 2a	SQ	2.5 × 10^7^ (Low dose)	14				
					5 × 10^7^ (High dose)	11				
CSU [159]	Autograft	Adipose tissue	Clinical study	IV	1 × 10^6^/kg (about 6 × 10^7^ for 60 kg)	Treated 10 Control 10	2 times	2 weeks	Favourable	None

*AD* atopic dermatitis, *CSU* chronic spontaneous urticarial, *SQ* subcutaneous administration, *IV* intravenous administration, *Treated* MSC therapy group, *Control* control group

Treatment with hUCB-MSCs significantly decreased the clinical severity, according to the Eczema Area and Severity Index (EASI) score, of 55% of patients. The hUCB-MSCs reduced the serum IgE levels and blood eosinophil counts. There was no serious adverse event. This clinical study was the first to confirm the efficacy and safety of allogeneic MSCs for AD. The improvement of clinical severity was dose-dependent. However, the laboratory values did not show a significant difference at the end of treatment. Three more clinical trials are ongoing in Phase 1, Phase 2 and Phase 1/Phase II [158].

### Mesenchymal Stem/stromal cell therapy (MSCT) in CU

#### Clinical trial in humans

There has been only one study of MSCT in humans with CU. In 2020, Özgül Özdemir et al. tried MSCT in the CU using autologous BM-MSCs as an experimental, open-label, single-centre clinical trial (Table 3). Ten patients were involved, and ten patients were included as a control group [159]. MSCs were administered intravenously at a dose of 1 × 10^6^ cells/kg (6 × 10^7^ cells for a body weight of 60 kg) two times at an interval of 2 weeks, and clinical changes were followed up for 6 months. Those with CSU at least 1 year earlier according to the EAACI/GA2 LEN/EDF/WAO guidelines, those unable to achieve disease control despite using omalizumab and/or cyclosporine for 6 months or longer, and those who had a weekly urticaria activity score (UAS7) greater than 20 were included in the study [160]. Patients with chronic inducible urticaria, AD, another underlying itchy skin disease, parasitic infection, or a history of malignancy were excluded. MSC treatment applied to refractory CSU patients was well tolerated, and no adverse effects were reported. Clinical responses were classified as well-controlled, partially responding and unresponsiveness. On day 14, two patients were well controlled, and eight patients partially responded, but in the first month, four patients continued to be partially responsive, and six were unresponsive. Three patients progressed to the well-controlled state at the sixth month, but two patients did not show clinical improvement. The first-, third- and sixth-month UAS7 scores of the group treated with MSCs significantly decreased compared to those of the control group.

The 14th-day frequencies of CD4 + IFNγ + and CD4 + Gata3 + cells in the treated group were significantly higher than those in the control group by flow cytometry analysis, and there was a significant difference between the MP and CP groups in terms of the TGF-β1 and IDO values measured on the 14th day. These immunological effects may have been due to the switching on of transient cytokine changes.

## Clinical viewpoints of mesenchymal stem/stromal cell therapy in atopic dermatitis and chronic urticaria

From the immunologic roles of MSCs, MSCT is expected to be symptomatic as well as a causative and even preventive treatment. In a clinical trial for AD [157], allografts of MSCs were well tolerated, without rejection (Table 3).

MSCs that were administered systemically by intravenous injection seemed to be localized to the lung, according to the results of an animal study. These MSCs gave favourable effects in CSU [159]. CSU is a systemic allergic disease, and it is suspected that MSCs act systemically.

AD is also a systemic disease that involves the skin of the whole body. The subcutaneous administration of MSCs locally improved AD. Local administration by subcutaneous injection showed systemic effects [157].

MSCT was effective in animal studies, which were performed on the basis of in vitro and in vivo studies in animal models, as expected. Similar results were predicted and have been achieved in humans. Although there are some differences between human diseases and animal models, future clinical trials in humans should be successful, based on the results of past animal studies and recent clinical trials in humans.

The routes of administration were subcutaneous and intravenous injection. Both seemed to be effective, as described above. However, subcutaneous injection seems to be safe considering side effects such as pulmonary embolism. In one group, intravenous administration was more effective than subcutaneous administration in an animal model of AD [132]. However, they chose subcutaneous administration in a subsequent clinical trial in human AD [157].

Although there has been just one clinical trial in AD and one clinical study in CU, the number (dose) of MSCs seems to be 5 × 10^7^ [Table 2]. The frequency of injection was a single dose or two times. In CSU, two repeated injections was successful. The interval between injections was 2 weeks for CSU. Both studies showed that the treatment was clinically effective and safe, with no severe adverse effects.

Most importantly, in a human study of MSCT in CSU, the clinical scores improved persistently for 6 months in some patients who were treated with MSCs, compared to recurrence in those who were treated with omalizumab, which is a new therapeutic biologic that is known to be very effective against CSU [159]. These results indicate that MSCs have great and broad potential as a new therapeutic modality for MSCs.

## Perspective

Mesenchymal stem/stromal cell therapy (MSCT) is a different therapeutic modality from previous pharmaceuticals or biologics. The nature of stem cells, including their characteristics and their relevant differences in effectiveness according to their source and their phenotypes and differentiation according to the immunologic environment, should be further investigated and considered for effective therapeutic application.

## Conclusion

Mesenchymal stem/stromal cell therapy is an unprecedentedly promising and potent therapeutic modality for allergic diseases in humans, especially for recalcitrant AD and CU. Further clinical trials and basic research in humans may be needed. Mesenchymal stem/stromal cell therapy will not only change the therapeutic landscape of human diseases but also improve our understanding of human biology from pregnancy to diseases such as malignancies, autoimmune diseases, degenerative diseases and allergic diseases through therapeutic trials.

## Data Availability

Not applicable.

## Abbreviations

CU: Chronic urticarial
CSU: Chronic spontaneous urticarial
SVF: Stromal vascular fraction
SVFC: Stromal vascular fraction cell
ASC: Adipose stem cell
ECM: Extracellular matrix
PRP: Platelet-rich plasma
